# Diet, Inflammation, and Infectious Diseases

**DOI:** 10.3390/nu15132891

**Published:** 2023-06-26

**Authors:** William B. Grant

**Affiliations:** Sunlight, Nutrition, and Health Research Center, P.O. Box 641603, San Francisco, CA 94164-1603, USA; wbgrant@infionline.net

This Special Issue, “Diet, Inflammation, and Infectious Diseases”, focuses on the potential of diet to modulate inflammation and infectious and chronic disease outcomes. The interaction of diet with gut integrity, microbiota, and metabolism and its effect on inflammation is also of interest. Six papers in this collection discussed diet. One paper looked at diet–microbiota interactions in inflammatory bowel disease [1], one looked at the modification of the gut microbiota via selected specific diets for people with Crohn’s disease (CD) [2], two focused on specific diets for treating CD [3,4], one investigated how a plant-based diet could reduce the prevalence of overactivated immunity for those with risk of metabolic syndrome [5], and one discussed diet with regard to HIV status [6]. In addition, one review discussed vitamin D and viral infections [7].

The review of diet–microbiota interactions in inflammatory bowel disease (IBD) by Sugihara and Kamada [1] noted that diet affects the composition of the gut microbiota, thereby playing a critical role in intestinal homeostasis. On the other hand, intestinal inflammation induces gut dysbiosis and may affect the use of dietary nutrients by host cells and the gut microbiota. The interaction of diet and the gut microbiota is perturbed in patients with IBD. In a healthy gut, the microbiota produce short-chain fatty acids, regulate bile acid metabolism, and produce vitamins. In IBD, these processes are attenuated, and the integrity of the mucosal and epithelial barriers is reduced. Important beneficial components of diet include fibers, good fats, tryptophan, and L-serine. Emulsifiers, found in processed foods, adversely affect the gut microbiota. Several modes of nutritional intervention were discussed regarding the gut microbiota in IBD.

Starz et al. [2] reviewed how selected specific diets can modify the gut microbiota in patients with one of the common IBDs, CD. The diets discussed include the low-FODMAP (fermentable oligosaccharides, disaccharides, monosaccharides, and polyols) diet, elimination diets, and the CDED (CD exclusion diet) with partial enteral nutrition. While some of the diets can reduce the symptoms of CD, they may also adversely affect the gut microbiota by reducing the intake of prebiotic substances, thereby negatively affecting the gut microbiota composition. 

Cantarelli et al. [3] published a comment on the Starz et al. review. They used the CDED in association with infliximab and methotrexate as a rescue therapy in a child affected by CD and chronic recurrent multifocal osteomyelitis who was resistant to optimized therapy. Both intestinal and bone symptoms remitted after the application of CDED. It appears that the diet may have acted on common microbiota-inciting agents that trigger both intestinal and bone inflammation, supporting the role of microbiota in the pathogenesis of IBD-associated extraintestinal manifestations. In a reply, Starz et al. [4] congratulated Cantarelli et al. and noted that there is additional research underway regarding the CDED.

For those interested in the topic of the gut microbiome and effects related to diet and disease, a review published in Nutrients in 2019 presents an excellent overview of the topic [8]. It reviews the role of diet quality, carbohydrate intake, fermentable FODMAPs, and prebiotic fiber in maintaining healthy gut flora.

Park and Zhang [5] studied the effect of a plant-based diet and physical activity in treating overactivated immunity with metabolic syndrome (MetS) risk. They categorized 40,768 participants in a Korean hospital cohort into four groups based on white blood cell (WBC) count, an index of innate immunity, and C-reactive protein (CRP), an index of inflammation. Daily intake of energy, carbohydrate, protein, and fat was not significantly different in the groups based on WBC counts and CRP. The results showed that a plant-based diet (PBD), physical activity, and non-smoking were related to lowering WBC counts and CRP, but a Western-style diet was linked to elevated CRP. A high PBD intake and smoking status interacted with immunity to influence MetS risk: a low PBD and current smoking were associated with a higher MetS risk in the H-WBC + H-CRP. 

Goosen et al. [6] presented findings regarding HIV and iron status with respect to nutritional and inflammatory status, anemia, and dietary intake in South African school children. Compared to HIV-negative counterparts, HIV-positive children reported significantly lower daily intake of animal protein, muscle protein, heme iron, calcium, riboflavin, and vitamin B_12_, and significantly higher proportions of HIV-positive children did not meet vitamin A and fiber requirements. Compared to iron-sufficient non-anemic counterparts, children with low iron stores reported a significantly higher daily intake of plant protein, a lower daily intake of vitamin A, and lower proportions of inadequate fiber intake. Along with best treatment practices for HIV, optimizing dietary intake in HIV-positive children could improve nutritional status and anemia in this vulnerable population.

It has become abundantly clear in the past couple of decades that the Western diet, with high amounts of animal products, such as red and processed meat as well as ultraprocessed foods with little fiber, is a major cause of chronic diseases. A recent article from Harvard reported findings regarding dietary patterns and food groups and risk of chronic diseases based on following health professionals for up to 32 years with use of food frequency questionnaires every 4 years [9]. They identified two dietary patterns associated with greatest risk reduction for diabetes: low insulinemic and low inflammatory. These two dietary patterns were associated with a 65% reduced risk of type 2 diabetes mellitus for 90th percentile vs. 10th percentile adherence. They were also associated with a ~40% reduction in major chronic disease. The food groups with highest risk were red and processed meats, French fries, and both low- and high-energy drinks. The food groups with highest risk reduction were coffee, wine, whole grains, fruit, and dark-yellow and leafy green vegetables.

Siddiqui et al. reviewed the evidence regarding the role of vitamin D in the immunity against viral infections [7]. They considered the effect on rhinovirus, influenza virus, respiratory syncytial virus, dengue virus, hepatitis C virus, HIV, and SARS-CoV-2. They discussed both innate immunity and adaptive immunity. The innate immune responses include chemotaxis, phagocytosis, and induction of cathelicidin and defensins. The adaptive immune response involves learning to recognize harmful viruses and deal with them through effects on B and T cells. In the discussion regarding influenza, they recognized that vitamin D was not the only reason why influenza rates are higher in winter than in summer. There are, indeed, other mechanisms involved. One is cold temperature. Eccles reviews a number of effects related to temperature and the success of common respiratory viruses [10]. Another is the release of nitric oxide from nitrate stores in the skin by solar UV radiation [11]. Nitric oxide has antimicrobial properties, and in that article, it was demonstrated to reduce COVID-19 deaths.

As the manuscript was accepted in August 2020, it was early in the COVID-19 pandemic, and the effect of serum 25-hydroxyvitamin D [25(OH)D] and vitamin D supplementation was not well understood by that time. There is now good evidence that vitamin D does reduce the risk of SARS-CoV-2 infection and subsequent COVID-19, and that early treatment with high-dose vitamin D can improve survival. An observational study was conducted based on data from veterans receiving treatment at US Veterans Administration Health facilities [12]. Patients who received vitamin D supplementation via prescription were compared to those who did not. Those who received vitamin D_3_ had a 28% reduced risk of COVID-19 [HR = 0.72, (95% CI 0.65, 0.79)] and a 33% reduced risk of mortality [HR = 0.67, (95% CI 0.59, 0.75)]. Since two important roles of vitamin D in reducing the risk of COVID-19, reducing the virus viability, and reducing risk of an overactive immune response including a cytokine storm, serum 25(OH)D concentrations have to be raised rapidly near the time of infection. To this end, supplementing with high-dose calcifediol [25(OH)D] raises 25(OH)D concentrations more rapidly than with cholecalciferol (vitamin D_3_). A pilot study conducted in Barcelona with calcifediol greatly reduced progression to the intensive care unit (ICU) and morality [13]. Of the 50 patients treated with calcifediol, one required admission to the ICU (2%), while of the 26 untreated patients, 13 required hospital admission (50%) *p* < 0.001.

This Special Issue has provided some interesting insight into the interaction of diet, inflammation, and infectious diseases.
